# Calcaneo-Stop: An Effective Surgical Technique to Correct Symptomatic Flexible Flatfoot in Children

**DOI:** 10.1055/s-0045-1804494

**Published:** 2025-04-11

**Authors:** Leonardo Lima de Almeida, Paulo Henrique Bortolin, Diego Polizello, Leonardo Rigobello Bataglion, José Batista Volpon

**Affiliations:** 1 Departamento de Ortopedia e Anestesiologia, Hospital das Clínicas, Faculdade de Medicina de Ribeirão Preto, Universidade de São Paulo, Ribeirão Preto, SP, Brasil

**Keywords:** adolescent, flatfoot, subtalar joint

## Abstract

**Objective** Flexible juvenile valgus flatfoot is a prevalent condition that typically resolves spontaneously but requires surgical treatment if associated with discomfort, pain, and physical limitation. However, certain surgical procedures are associated with higher morbidity, as they involve osteotomies and arthrodesis. In this context, extra-articular talocalcaneal arthroereisis is an option, because it is a cost-effective, low-morbidity, and efficient method of treatment. The aim of the present study is to report the outcomes of the treatment of flexible juvenile valgus flatfoot using the calcaneo-stop technique.

**Methods** Pre-adolescent patients with severe or symptomatic flexible juvenile valgus flatfoot were surgically treated using the calcaneo-stop technique (talocalcaneal arthroereisis). The outcomes were evaluated clinically and radiographically, assessing Meary angles, talonavicular coverage, talus-metatarsal alignment, Moreau-Costa-Bertani angle, calcaneal pitch, and the percentage of talus head coverage by the navicular.

**Results** A total of 23 individuals (44 feet) were evaluated and operated on, with ages ranging from 7 to 13 years (mean: 11) and a mean follow-up of 28 months. There was marked improvement or disappearance of symptoms in 90% of the patients. The complication rate was of 13.6%, primarily associated with localized pain at the surgical site. All radiographic parameters improved significantly ( *p* < 0.001), with values approaching normality in most cases.

**Conclusion** Improvements in both clinical and radiographic parameters suggest that talocalcaneal arthroereisis corrects deformities with a low complication rate.

## Introduction

Flexible flatfoot is characterized by the absence of or significant reduction in the medial longitudinal arch, associated with an increased valgus of the ankle during weight bearing. It is a common condition in children, but it is self-limiting and has a strong tendency for spontaneous regression. 1 However, a minority of individuals may experience complete failure in the development of the medial plantar arch, resulting in permanent flatfoot throughout life. In adulthood, if the condition is severe, there may be functional limitations, pain, and an increased risk of developing osteoarthritis. 2

In the juvenile age group, flatfoot can be associated with symptoms such as fatigue after usual efforts, cramps, pain, and discomfort when attempting to fit the unconventional foot shape into standard footwear. 3 These complaints tend to be exacerbated with excess weight, ligament laxity, or Achilles tendon shortening. 4

Conservative interventions, such as the use of insoles, despite their widespread use, lack scientific evidence supporting their effectiveness. 5 6 As a result, several surgical procedures have been developed to correct symptomatic flexible flatfoot in immature individuals. These techniques can be grouped into soft-tissue procedures, 7 osteotomies, 8 9 10 or localized arthrodesis. 4 Approaches that exclusively target musculotendinous and ligamentous structures provide temporary corrective effects due to tissue compliance. Osteotomies yield favorable outcomes, but they require longer periods of immobilization and recovery. Arthrodesis has limited indications, as it requires joint destruction to achieve correction. 11

Another surgical option is arthroereisis (from the Greek *arthroereisis* , in which *arthros* means joint, and *ereisis* , the action of supporting or sustaining). These procedures aim to preserve the joint providing mechanical stabilization without limiting functional movements. They are interesting for their potential in correcting and preventing severe deformities. 12 When applied to flatfoot, such techniques show simplicity, efficiency, low morbidity, and prompt recovery, making them suitable for young and healthy patients. 13

The origins of arthroereisis in the foot can be attributed to Grice, 14 in 1952, who devised an extra-articular arthrodesis procedure by inserting a bone graft into the tarsal sinus, attempting to correct valgus deformities in children affected by poliomyelitis sequelae. In 1990, Crawford et al. 12 employed temporary metal staples in the subtalar joint to correct valgus feet in individuals with spastic cerebral palsy. The primary objective was to restore the medial plantar arch by partially restricting subtalar joint motion, rather than completely blocking it. Later, the arthroereisis effect was achieved through the insertion of implants into the tarsal sinus, or by blocking excessive calcaneal eversion using a screw placed laterally to the tarsal sinus, under the lateral process of the talus. 15

Subtalar implants within the tarsal sinus were initially introduced in 1977, utilizing a silicone elastomer block to maintain the optimal position of the joint. 16 Since then, a wide variety of implants have been developed for this purpose, encompassing various shapes such as plugs, spacers, cones, screws, and cylinders. The materials include elastomer, polyethylene, titanium, and bioabsorbable poly-L-lactic acid. 3

According to De Pellegrin, 15 the insertion of an extra-articular screw into the calcaneus, adjacent to the lateral process of the talus, was initially described by Álvarez 17 to achieve the effect of arthroereisis, and later modified by Pisani. 18 The technique temporarily blocks excessive pronation of the subtalar joint, enabling the lateral column to grow and occupy the space without pressure.

Currently, this technique is widely used in Europe. 13 15 18 19 20 In Brazil, arthroereisis with a screw in the calcaneus remains, to some extent, unfamiliar to most of the orthopedists, and the publications are on the treatment of neuropathic flatfoot. 21

The objectives of the present study were to analyze the results of correcting severe symptomatic flexible valgus flatfoot in the preadolescent age group using the calcaneo-stop technique, and to contribute to a wider dissemination of this method.

## Materials and Methods

**Fig. 1:**
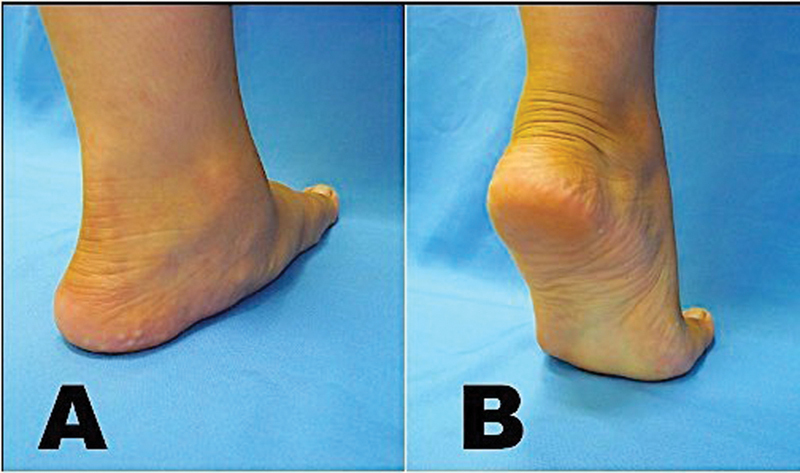
Tiptoe standing test to demonstrate foot flexibility. ( **A** ) On weight bearing, the medial arch collapses. ( **B** ) The heel turns into varus and the medial arch reappears.

The current retrospective study was designed to evaluate the outcomes of the surgical treatment in preadolescent patients with symptomatic flexible valgus flatfoot. The study was approved by the institutional Ethics Committee (CAAE: 77119524.8.0000.5440). The sample included individuals of both sexes presenting severe, flexible, idiopathic valgus flatfoot submitted to the calcaneo-stop technique at our institution. The flexibility of the foot was assessed based on reversibility of the deformity with and without weight bearing, the tiptoe standing test ( Fig. 1 ), and on the result of the Jack test.

Data were collected from medical records, and the assessment included the resolution of the symptoms, the satisfaction of the patients and their families, as well as pre- and postoperative radiographic parameters. The study included cases with complete documentation and informed consent, involving surgeries performed between 2017 and 2022, with an average follow up of 28 months (range: 12-65). We excluded cases of lower limb mechanical axis deviation, concurrent foot deformities or stiffness, prior foot surgery or fractures, or underlying local or systemic diseases (Down syndrome, Ehlers-Danlos syndrome, etc.).

### Operative Procedure

**Fig. 2:**
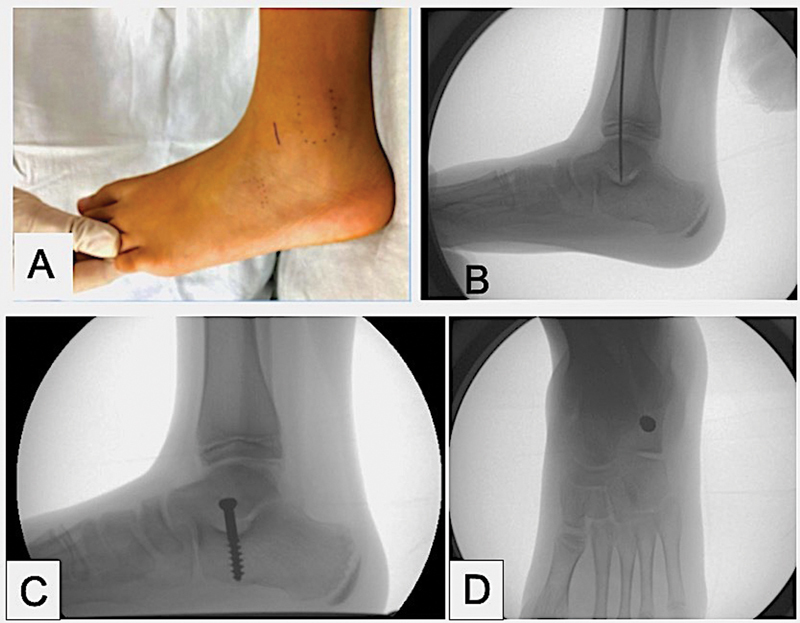
Surgical steps for calcaneal screw insertion. ( **A** ) Cutaneous demarcation of the surgical incision over the sinus tarsi. ( **B** ) After blunt dissection, the entrance of the sinus tarsi was exposed, and a guide K wire was vertically inserted into the calcaneus ( **C** ) Screw positioning in lateral view. ( **D** ) Screw positioning in anteroposterior view.

Under anesthesia, with the knee extended, the foot was forced into dorsiflexion to assess the degree of heel valgus and to check for associated equinus. Palpation was used to identify the sinus tarsi, and a skin incision measuring approximately 2.0 cm in length was made over it ( Fig. 2A ). Careful blunt dissection was performed to expose the fat pad and the short extensor muscle, ensuring the sensory branch of the fibular nerve remained undamaged. Under fluoroscopy, the foot was forced into supination, enabling the identification and exposure of the flat surface in the lateral region of the calcaneus, serving as the entry point to the sinus tarsi. A Kirschner wire (K wire) with 2.0 mm in diameter was perpendicularly inserted into the calcaneus bone surface, outside of the sinus tarsi, and its position was confirmed using fluoroscopy ( Fig. 2B ). Subsequently, the K wire was removed, but the hindfoot was maintained in varus position. The entry point of the wire was identified, the hole was enlarged using a 3.2-mm drill bit, and a partially threaded 6.5-mm cancellous stainless-steel screw (DePuy Synthes, Raynham, MA, United States) with 30 to 40 mm in length was inserted ( Fig. 2C,D ). The depth of the screw was adjusted to ensure its head was positioned beneath the lateral process of the talus, thereby correcting the malalignment in valgus of the hindfoot. If under- or overcorrection occurred, the screw was respectively advanced or retracted until the desired alignment was achieved. In certain cases, alternative implants, such as those of 7.0-mm cannulated cortical screws (DePuy Synthes), or fully-threaded screws were used. It should be noted that the available literature does not indicate any significant impact of these implant variations on the outcomes of the procedure. If residual equinus was present, a Hoke-type lengthening of the Achilles tendon was carried out, followed by the application of a long leg plaster cast splint.

### Postoperative Care

Postoperatively, all patients were immobilized for 7 days, after which weight bearing was gradually reintroduced. In cases requiring equinus correction, patients wore a walking cast for an additional two weeks before transitioning to regular footwear.

A standardized physiotherapy protocol was not implemented, nor was the use of orthotics or modified footwear recommended. Around three weeks after surgery, once the scars had fully healed, the patients were advised to gradually resume physical activities and sports, provided postoperative pain had subsided.

#### Clinical Evaluation

The patient's medical record was accessed to compare the pre- and postoperative findings. Complications resulting from the surgical procedure, along with symptoms observed postoperatively that were not present preoperatively, were taken into consideration, such as pain, discomfort, and esthetic improvement. 22

### Radiographic Evaluation

**Fig. 3:**
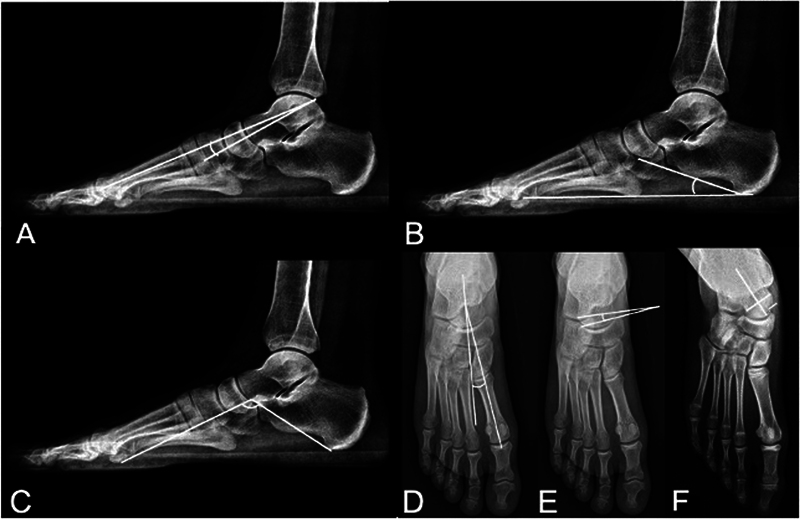
X-Ray evaluation of the foot deformities. ( **A** ) Meary's angle: measured in lateral view, it represents the angle formed between the long axis of the talus and the long axis of the first metatarsal (normal range: 5°–15°). ( **B** ) Calcaneal pitch: measured in lateral view, it represents the angle formed between the inferior surface of the plantar cortex of the calcaneus and the ground (normal range: 15°–25°). ( **C** ) Moreau-Costa-Bertani's angle: determined by tracing a line from the inferior surface of the medial sesamoid to the inferior surface of the talonavicular joint. Another line is traced from this point to the posterior tuberosity of the calcaneus (normal range: 115°–125°). ( **D** ) Talus-metatarsal angle: measured in anteroposterior view, it represents the angle between the long axis of the talus and the long axis of the first metatarsal (normal values range: 0°–5°). ( **E** ) Coverage angle of the talonavicular joint: measured in anteroposterior view, it represents the angle formed by the medial and lateral articular surfaces of the talus and the articular surface of the navicular bone (normal range: 0°–7°). ( **F** ) Percentual coverage of the talus head is the percentage of the articular surface of the talus head that is covered by the navicular (normal: ≥ 90%).

The pre and the latest postoperative images were evaluated in anteroposterior and lateral views, under weight bearing. The following angles and measurements were assessed: Meary's, calcaneal pitch, Moreau-Costa-Bertani's, alignment between the long axis of the talus with the first metatarsal, talonavicular angular coverage, and talonavicular percentual coverage. 23 24 Fig. 3 shows the angles used, how to trace them, and their normal ranges. All collected data were grouped and presented in Table 1 .

**Table 1:** Study sample data

Identification	Age	Follow-up	Calcaneal pitch		Talonavicular angle coverage		Talonavicular percentual coverage		Meary's angle		Talus-first metatarsal angle		Moreau-Costa-Bertani's angle		Pain		Complications
	(years)	(month)	Pre	Post	Pre	Post	Pre	Post	Pre	Post	Pre	Post	Pre	Post	Pre	Post	
F1	9	20	4.5	11.8	45.6	22.4	50.6	24.8	-32.6	-5.5	25.3	2.7	159	140.8	Yes	No	No
F2	9	20	7.7	14	44.8	21.8	47.7	24.2	-23	-5.6	14.3	7.4	148.2	135.3	Yes	No	No
F3	13	34	6	16.7	36.6	16.5	40.6	18.3	-24	-13.7	12.4	3.4	142.4	132.4	SIM	No	No
F4	13	28	8.6	17.3	23.9	11.6	26.5	12.8	-22	-8.6	13.3	1.4	142.8	127.8	Yes	No	No
F5	9	34	2	4	56.1	24.7	62.3	27.4	-18.1	1.7	18.6	9.7	143.4	135	Yes	No	No
F6	9	34	-4	1.5	45.9	25	51	27.7	-18	-4.6	20.3	1.2	147.6	135.8	Yes	No	No
F7	11	40	2.6	10.5	45.8	18.7	50.8	20.7	-17.2	-0.8	17.1	5.5	146.1	133.1	Yes	No	Chronic pain
F8	11	40	1	8.7	44.1	15.1	49	16.7	-22	-1.5	16.4	5	141.6	134.9	Yes	Yes	Chronic pain
F9	12	30	0.5	4.3	41.3	1	45.8	1.1	-17.8	-12.8	16.8	5.2	144.2	134	Yes	No	No
F10	12	30	0.5	6	50.4	23.3	56	25.8	-23.2	-9.8	36.1	17.3	151.5	135.3	Yes	No	No
F11	12	37	10.5	13.8	16	11.3	17.7	12.5	-6.7	6.3	25	14.3	130.3	115.6	Yes	No	No
F12	12	37	10.8	12.1	39.5	16.8	43.8	18.6	-14.6	1.6	36.2	19	134.7	119.5	Yes	No	No
F13	11	21	6.3	9.5	39	21.5	43.3	23.8	-30.6	-12.5	23.5	3	151.7	142.5	Yes	No	No
F14	11	21	9	7.8	11.9	9.8	13.2	10.8	-12.5	-7.7	6	7.8	141.6	137.7	Yes	No	No
F15	11	39	11	18	40.2	1.1	44.6	1.2	-10.7	13.3	24.1	6.5	138.7	118.6	Yes	No	No
F16	11	39	11.6	18.4	42.9	2.3	47.6	2.5	-12.5	-3.5	29.4	3.5	140	127	Yes	No	No
F17	12	17	4.7	5.1	32.9	20.2	36.5	22.4	-22.1	-13.5	12	12.7	152.6	144.2	Yes	No	Undercorrection
F18	12	17	3.3	9.6	43	27.6	47.7	30.6	-28	-4.5	25.5	1.9	153.4	131.2	Yes	No	No
F19	11	47	9.6	15	38.8	12.8	43.1	14.3	-19	-5.3	19.2	9.3	151.2	129.5	Yes	No	No
F20	11	47	6.5	16.8	32	11.9	35.5	13.2	-19.7	-2	22.4	12.9	148.1	129.9	Yes	No	No
F21	9	65	3.9	17.3	42.8	15.5	47.5	17.2	-20.6	-4.4	16.8	8.6	144.8	130.4	Yes	No	No
F22	9	65	3.3	13.3	46.9	22.7	52.1	25.2	-30	-7.1	20.1	8.1	158	137.2	Yes	No	No
F23	9	6	3.6	7.9	26	20.4	28.8	22.6	-22.4	-12.2	4.4	2.3	148.4	141	Yes	Yes	Chronic pain
F24	16	33	10.1	12.2	35.6	31.7	39.5	35.2	-9.5	-6	14.8	13	131.4	132	Yes	No	No
F25	16	33	8.1	14.1	30.1	24.8	33.4	27.5	-14.6	-5.2	16	11	136.9	128.1	Yes	No	No
F26	11	32	7.9	15.6	41.6	15.1	46.2	16.7	-18.7	-5.7	13.6	1.1	142.9	137.6	Yes	No	No
F27	11	32	10.6	14.8	39.6	6.9	44	7.6	-13.4	-6.5	13.5	1.6	140.3	137.4	Yes	No	No
F28	7	11	7	12.7	41.6	7.1	46.2	7.8	-9.5	10.6	12.5	9.2	140.9	124.6	Yes	No	No
F29	7	11	6.7	8.2	33.8	4.5	37.5	5	-10.5	10.1	14.1	14.4	138.7	128	Yes	No	No
F30	13	26	13.4	20.1	32.5	12.1	36.1	13.4	-12.9	4.8	36.6	16	134.8	121.8	Yes	No	No
F31	13	26	19.3	20.6	36.4	16.6	40.4	18.4	-11.4	4.1	35.3	12.7	124.7	117.2	Yes	No	No
F32	10	6	9.7	16.2	26.4	14.7	29.3	16.3	-9.6	0.8	13.7	11.2	133.5	122.1	Yes	No	No
F33	10	6	14.4	17.3	20.8	2.3	23.1	2.5	-10.1	1.5	10.2	5	127.6	118	Yes	No	No
F34	11	47	5.7	8.1	27.7	23.4	30.7	26	-17.7	3.5	16	11.8	142.2	128.7	Yes	No	No
F35	11	47	7.1	8.7	23.8	18	26.4	20	-18.7	3.9	14	7	143.8	127.1	Yes	No	No
F36	13	20	10.4	17.4	25.2	9.9	28	11	-11.2	-1	4.2	4.7	133.2	119.1	Yes	No	No
F37	13	20	11.8	18.2	25.8	15.5	28.6	17.2	-13.6	-2.5	3.2	0.1	136.8	124.3	Yes	No	No
F38	12	12	3	12.7	43	23.9	47.7	26.5	-24	-3.5	22	9.8	158	126.5	Yes	No	No
F40	14	12	4.3	7.5	32.1	4.4	35.6	4.8	-21.7	-14.3	19.1	6.8	154.2	147.5	Yes	No	Undercorrection
F41	14	12	5.5	11.1	36.7	5	40.7	5.5	-19.2	-10.4	23.2	9.8	150.1	143.2	Yes	No	Undercorrection
F42	7	26	3.3	9.2	42	16.2	46.6	18	-13.3	-7.1	14.4	4.4	144.3	134.4	Yes	No	No
F43	7	26	4.1	10.1	40.7	13.3	45.2	14.7	-11.9	-6.6	13.2	3.9	142.1	129.1	Yes	No	No
F44	12	34	2.2	16.4	41.2	20.2	45.7	22.4	-23.1	-9.4	16.8	4.4	151.1	139.1	Yes	No	No

**Abbreviations:** Pre, preoperative; Post, postoperative.

### Statistical Analysis

The Shapiro-Wilk test was used to evaluate the normality of the sample distribution and determine a parametric distribution, with statistical significance set at *p* < 0.05. The paired *t* -test was used to assess the significance of the surgical intervention and improvement in radiological outcomes, with *p* set at < 0.001. Analyses were conducted using the RStudio Desktop Pro (Posit, PBC, Boston, MA, United States) software, version 2023.03.0.

## Results

A total of 23 patients (44 feet), aged between 7 and 13 (mean: 11) years, were considered eligible for the study. The minimum follow-up was 12 months, and the maximum was 5 years (65 months), with a mean of 28 months.

**Fig. 4:**
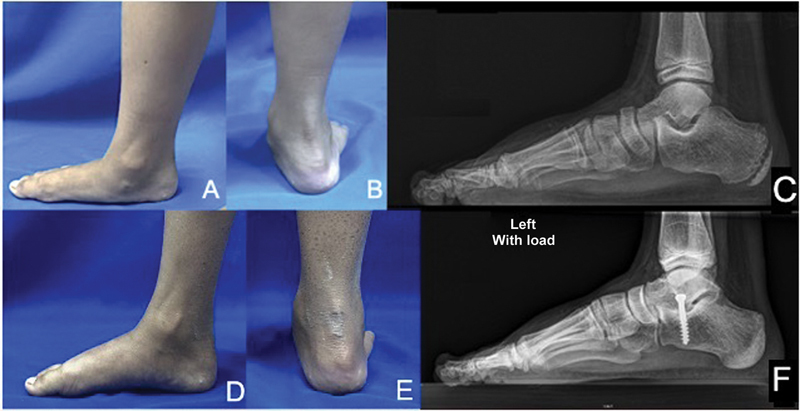
Pre ( **A,B** ) and postoperative ( **C,D** ) clinical appearance of a 10-year-old boy who underwent surgery to correct a severe valgus flat foot. Significant improvement was observed in both the ankle alignment and foot arch. In this case, in addition to the calcaneo-stop procedure, a percutaneous lengthening of the Achilles tendon was performed to correct the equinus. ( **E** ) Preoperative X-ray in lateral view performed in 2019. ( **F** ) Postoperative X-ray in lateral view performed in 2022.

The clinical outcomes were satisfactory, addressing both esthetic concerns and alleviating pain. In total, 90% of the patients reported improvement or complete resolution of symptoms and pain relief compared to their preoperative condition. The rate of complications was of 13.6%, and they occurred in 6 cases: 3 with chronic pain at the surgical site, and 3 with incomplete correction of the deformity. In two cases, the pain was resolved with local corticosteroid injections, while, in the other case, the screw was removed, resulting in reduced pain – though not fully resolved. Implant removal was not necessary for the other patients. There were no occurrences of infection, wound healing issues, or implant breakage. All radiographic parameters exhibited significant postoperative improvement ( Fig. 4 ).

**Fig. 5:**
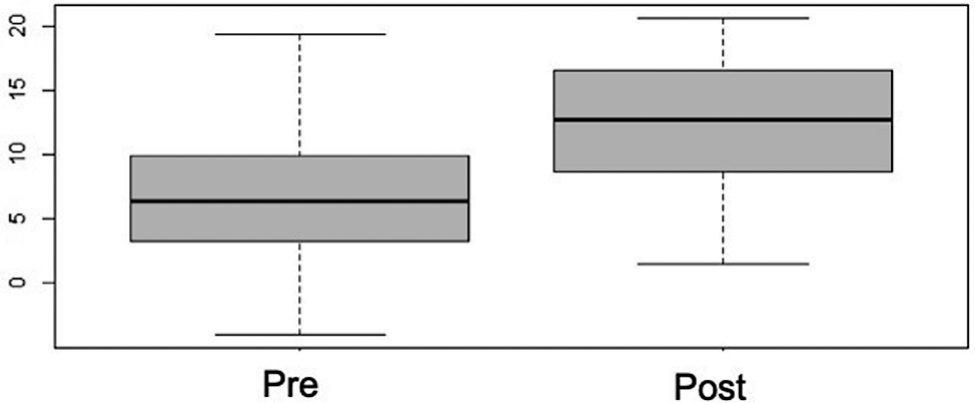
Box plot distribution for the pre and postoperative calcaneal pitch angle. There was a significant postoperative increase, and the angle reached normal ranges ( *p* < 0.001).

**Fig. 6:**
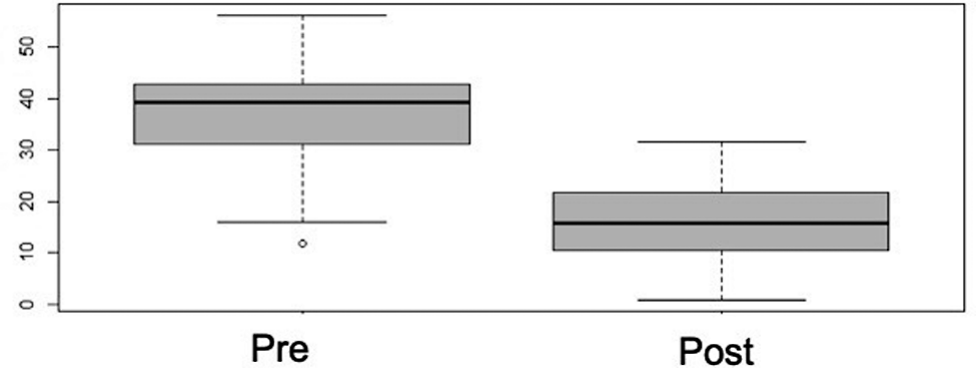
Box plot distribution for pre and postoperative talonavicular angle. There was significant postoperative improvement, and the angle almost reached normal ranges ( *p* < 0.001).

**Fig. 7:**
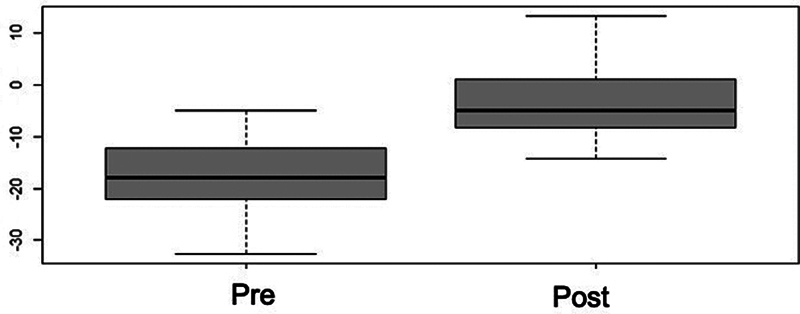
Box plot distribution for the pre and postoperative Meary's angle. There was significant postoperative improvement, and the angle was slightly below normal parameters ( *p* < 0.001).

The calcaneal pitch showed a significant increase of 87%, with a preoperative mean value of 6.61° (standard deviation [SD]: 0.65°) and a postoperative value of 12.41° (SD: 0.60°) ( Fig. 5 ). The talonavicular coverage exhibited a significant improvement, of 136%, as evidenced by the *t* -test ( *p* < 0.001). The final mean values of 15.43° (SD: ± 1.15°) remained slightly higher than the reference values ( Fig. 6 ). The Meary's angle showed a strong correlation in the *t* -test ( *p* < 000.1) ( Fig. 7 ), with a significant improvement of 383%, in the talonavicular alignment of the sagittal axis. However, the mean final value of -3.64° (SD: ± 1.01°) was still not within the normal range.

**Fig. 8:**
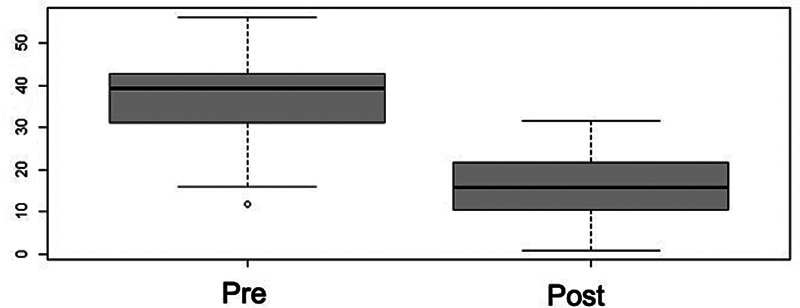
Box plot distribution for the pre and postoperative talus-metatarsal angle. There was significant postoperative improvement, and the angle almost reached normal ranges ( *p* < 0.001).

The alignment of the long axis of the talus with the first metatarsal in the coronal plane showed improvement of 141%, a significant difference in the *t* -test ( *p* < 0.001) ( Fig. 8 ). The mean preoperative value was of 18.12° (SD: ± 1.22°) and, postoperatively, it was of 7.5° (SD: ± 0.78°).

**Fig. 9:**
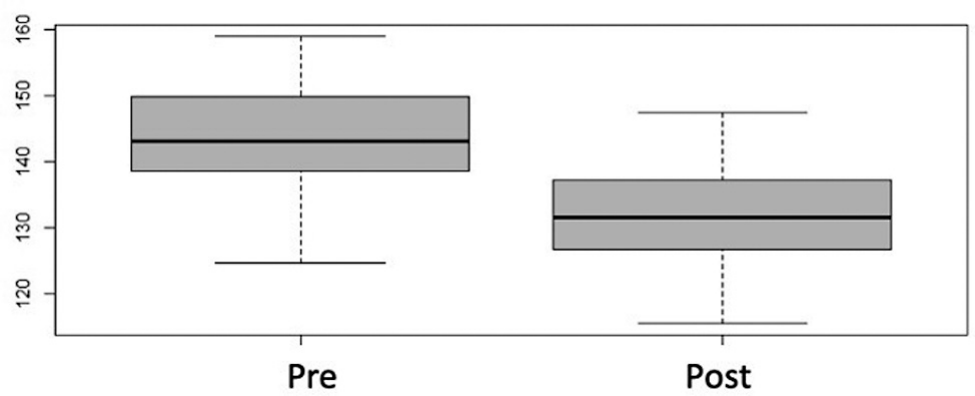
Box plot distribution for the pre and postoperative Moreau-Costa-Bertani's angle. There was a significant postoperative increase, and the angle reached normal ranges ( *p* < 0.001).

**Fig. 10:**
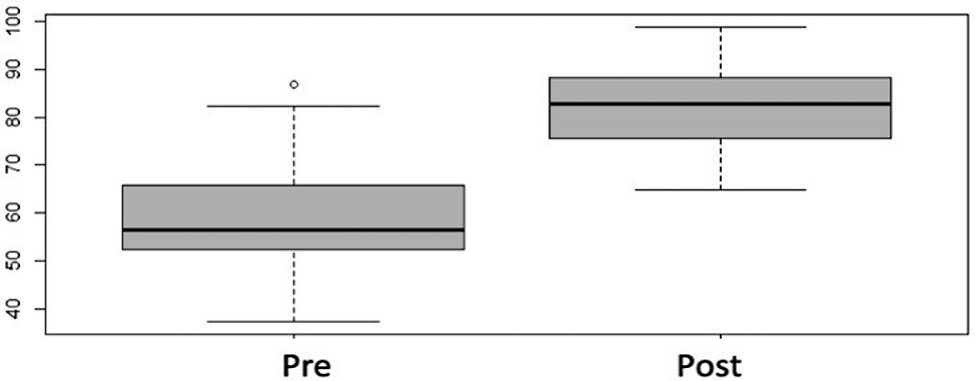
Box plot distribution for the pre and post-operative talonavicular coverage percentage. There was significant postoperative improvement, and the coverage almost reached normal ranges ( *p* < 0.001).

The Moreau-Costa-Bertani's angle showed improvement in the measured parameters ( *p* < 0.001). Although the values did not reach normality, with a preoperative mean of 143.8° (SD: ± 1.22°) and a postoperative mean of and 131.21° (SD: ± 1.19°), an improvement of 9% in the formation of the plantar arch was observed ( Fig. 9 ). The talonavicular coverage percentage showed significant improvement of 38% as evidenced by the *t* -test analysis ( *p* < 0.001). The preoperative mean value of 59.7% (SD: ± 1.54%) increased to 82.8% (SD: ± 1.28%), with a significant improvement in the relationship between the hindfoot and midfoot ( Fig. 10 ).

## Discussion

In the present case series, the treatment of the severe idiopathic pediatric flatfoot using the calcaneo-stop technique demonstrated effective correction of the deformity with minimal complications. The surgical procedure is technically straightforward and yields positive clinical and radiographic outcomes.

Although the exact cause of flatfoot remains a topic of debate, 7 25 if left untreated, it can lead to functional limitations in adulthood. Progressive subluxation and excessive loading of the triple joint complex and the spring ligament may occur, ultimately leading to late articular degeneration. This overloading is clinically responsible for fatigue, pain, and mechanical insufficiency.

The extra-articular technique known as calcaneo-stop involves the insertion of a screw into the calcaneus, effectively blocking excessive talus-calcaneus pronation and subsequent subluxations. 17 Moreover, research 15 26 27 suggests that, besides joint restriction, proprioceptive mechanisms influence gait kinetics. Unlike osteotomies, which immediately alter joint relationships and load distribution, arthroereisis induces an early correction of the deformities, followed by a late gradual three-dimensional articular reorganization over several months, leading to articular remodeling.

Lateral column lengthening osteotomies and medialization of the calcaneus are accepted treatments for symptomatic flexible flatfoot in the immature foot, with reported satisfactory results of approximately 90%. 8 9 10 However, these procedures are more invasive, require longer recovery time, and impose activity restrictions for weeks. In contrast, arthroereisis results in a fast rehabilitation, enabling the patient to walk shortly after surgery. 13 17 27 28 Nevertheless, in the present series, 42% of the patients underwent Achilles tendon lengthening and required plaster immobilization for up to 21 days. For the remaining patients, weight bearing was allowed on the seventh postoperative day. Conversely, some authors 15 do not routinely release the triceps surae, even in the presence of equinus, arguing that the postoperative equinus can be reversed with physiotherapy.

While clinico-radiographic dissociation exists, we employed angular parameters to evaluate and enhance the objectivity and reproducibility of the results. Nonetheless, these variables may not directly correlate with the symptoms and severity of the foot. Moreover, establishing the most significant signals for the diagnosis and determining the precise threshold to define a foot as a flat remains challenging. The relevance of radiographic measures and flatfoot diagnosis is still under debate. 23

Our radiographic assessments revealed improvement in hindfoot and midfoot parameters over a mean follow-up period of 28 months, which is consistent with the findings of previous studies. 19 27 28 The clinical outcomes were deemed satisfactory, with 90% of the patients reporting no pain. The complication rate was 13.6%, but they were minor complications, primarily attributed to pain that showed improvement following analgesic treatment. Our data and complication rates are similar to those reported in other studies. 19 20 27

Determining the optimal time for implant removal without compromising correction remains uncertain in the calcaneo-stop technique. Some authors 11 29 suggest removal after 2 years, during which joint remodeling and proprioceptive reflex incorporation occur. However, establishing a removal protocol in the present study was challenging due to high hospital service demands. As a result, most of the patients retained the implant for up to 5 years without experiencing overcorrection, pain, or breakage. The radiographic images showed no evidence of any impression on the talus secondary to prolonged contact at the implant interface. However, we did not precisely evaluate this parameter due to limited symptoms.

The current study has limitations, including a restricted sample size. Moreover, we faced challenges in quantifying the subjective and variable nature of pain and discomfort in a pediatric population and in establishing a correlation between the radiographic parameters and the clinical findings of the flatfoot.

Overall, our impression is that the calcaneo-stop technique seems to be an adequate option to treat symptomatic flexible flatfoot in juvenile patients, according to major studies. 30 31 It offers potential correction of the initial deformity and consistent improvement in clinical and radiographic parameters. Furthermore, the technique can be reversed through screw removal if necessary, and it does not compromise the use of other techniques in cases of failure. There are still questions regarding joint remodeling and possible long-term repercussions; however, the technique seems promising.
