# Supplementary material for: Calcaneo-Stop: An Effective Surgical Technique to Correct Symptomatic Flexible Flatfoot in Children
Source: Rev Bras Ortop (Sao Paulo). 2025 Apr 11;60(1):s00451804494. doi: 10.1055/s-0045-1804494 (PMC12020559; doi:10.1055/s-0045-1804494)
Supplement: Related texts

# Research article: Calcâneo-stop: Uma técnica cirúrgica eficaz para a correção do pé plano valgo flexível sintomático em crianças

**Keywords:** adolescente, articulação talocalcânea, pé chato

## Abstract

**Objetivo** O pé plano valgo juvenil flexível é uma doença prevalente que, de modo geral, se resolve de forma espontânea. No entanto, requer tratamento cirúrgico se associado a desconforto, dor e limitação física. Alguns procedimentos cirúrgicos, porém, são associados a maior morbidade, pois envolvem osteotomias e artrodese. Nesse contexto, a artrorrise talocalcânea extra-articular é uma opção por ser um método de tratamento barato, de baixa morbidade e eficaz. O objetivo deste estudo é relatar os resultados do tratamento do pé plano valgo infantil flexível usando a técnica calcâneo-stop.

**Métodos** Pacientes pré-adolescentes com pé plano valgo flexível grave ou sintomático foram submetidos ao tratamento cirúrgico usando a técnica calcâneo-Stop (artrorrise talocalcaneana). Os resultados foram avaliados clínica e radiograficamente, incluindo os ângulos de Meary, a cobertura talonavicular, o alinhamento tálus-metatarso, o ângulo de Moreau-Costa-Bertani, o *pitch* calcâneo e a porcentagem de cobertura da cabeça do tálus pelo navicular.

**Resultados** No total, 23 pacientes (44 pés) foram avaliados e operados. A idade dos pacientes variou de 7 a 13 anos (média: 11) anos, com acompanhamento médio de 28 meses. Houve melhora acentuada ou desaparecimento dos sintomas em 90% dos pacientes. A taxa de complicação foi de 13,6%, associada principalmente à dor localizada no sítio cirúrgico. Todos os parâmetros radiográficos melhoraram significativamente ( *p* < 0,001), com valores próximos da normalidade na maioria dos casos.

**Conclusão** Melhoras nos parâmetros clínicos e radiográficos sugerem que a artrorrise talocalcânea corrige deformidades com baixa taxa de complicações.

## Introdução

O pé plano flexível é caracterizado pela ausência ou redução significativa do arco longitudinal medial associadas a um aumento do valgo do tornozelo durante a sustentação de peso. É comum em crianças, mas é autolimitado e tem forte tendência à regressão espontânea. 1 No entanto, alguns indivíduos podem apresentar falha completa no desenvolvimento do arco plantar medial, o que resulta em pé plano permanente ao longo da vida. Na idade adulta, a doença grave pode causar limitações funcionais, dor e aumentar o risco de osteoartrite. 2

Na faixa etária juvenil, o pé plano pode estar associado a sintomas como fadiga com os esforços habituais, cãibras, dor e desconforto ao tentar adaptar o formato não convencional do pé ao calçado padrão. 3 Essas queixas tendem a ser exacerbadas em pacientes com excesso de peso, frouxidão ligamentar ou encurtamento do tendão calcâneo. 4

Intervenções conservadoras, como o uso de palmilhas, apesar de generalizadas, carecem de evidências científicas que sustentem sua eficácia. 5 6 Assim, vários procedimentos cirúrgicos foram desenvolvidos para corrigir o pé plano flexível sintomático em indivíduos imaturos. Essas técnicas podem ser agrupadas em procedimentos de tecidos moles, 7 osteotomias, 8 9 10 ou artrodese localizada. 4 Abordagens que visam exclusivamente estruturas musculotendíneas e ligamentares têm efeitos corretivos temporários devido à complacência do tecido. Osteotomias produzem resultados favoráveis, mas requerem períodos mais longos de imobilização e recuperação. A artrodese tem indicações limitadas, pois a correção requer destruição articular. 11

Outra opção cirúrgica é a artrorrise (do grego *arthroereisis* , em que *arthros* significa “articulação”, e *ereisis* , “ação de suportar ou sustentar”). Esses procedimentos visam preservar a articulação, e proporcionam estabilização mecânica sem limitar os movimentos funcionais. São interessantes devido à possibilidade de correção e prevenção de deformidades graves. 12 Aplicadas ao pé plano, estas técnicas são simples, eficientes, e apresentam baixa morbidade e rápida recuperação, sendo adequadas para pacientes jovens e saudáveis. 13

As origens da artrorrise no pé podem ser atribuídas a Grice, 14 que, em 1952, idealizou uma artrodese extra-articular por meio da inserção de um enxerto ósseo no seio tarsal, em uma tentativa de corrigir deformidades em valgo em crianças com sequelas de poliomielite. Em 1990, Crawford et al. 12 empregaram grampos metálicos temporários na articulação subtalar para a correção de pés valgos em indivíduos com paralisia cerebral espástica. O objetivo primário era restaurar o arco plantar medial ao restringir parcialmente o movimento da articulação subtalar, em vez de bloqueá-lo por completo. Mais tarde, o efeito de artrorrise foi alcançado por meio da inserção de implantes no seio tarsal ou bloqueando a eversão excessiva do calcâneo com um parafuso colocado lateralmente ao seio tarsal, sob o processo lateral do tálus. 15

Os implantes subtalares no seio tarsal foram introduzidos em 1977, utilizando um bloco de elastômero de silicone para manter a posição ideal da articulação. 16 Desde então, diversos implantes foram desenvolvidos com esse propósito e com vários formatos, como plugues, espaçadores, cones, parafusos e cilindros. Os materiais incluem elastômero, polietileno, titânio e ácido poli-L-láctico bioabsorvível. 3

De acordo com De Pellegrin, 15 a inserção de um parafuso extra-articular no calcâneo, adjacente ao processo lateral do tálus, foi descrita pela primeira vez por Álvarez 17 para obter o efeito de artrorrise e, posteriormente, modificada por Pisani. 18 A técnica bloqueia temporariamente a pronação excessiva da articulação subtalar, o que permite que a coluna lateral cresça e ocupe o espaço sem pressão.

Hoje, esta técnica é amplamente utilizada na Europa. 13 15 18 19 20 No Brasil, a artrorrise com parafuso no calcâneo ainda é, de certa forma, desconhecida pela maioria dos ortopedistas, e as publicações visam o tratamento do pé plano neuropático. 21

Os objetivos deste estudo foram analisar os resultados da correção do pé plano valgo flexível sintomático grave na faixa etária pré-adolescente com a técnica calcâneo-stop e contribuir para uma maior divulgação deste método.

## Materiais e Métodos

Este estudo retrospectivo foi delineado para avaliar os resultados do tratamento cirúrgico em pacientes pré-adolescentes com pé plano valgo flexível sintomático. O estudo foi aprovado pelo Comitê de Ética institucional (CAAE: 77119524.8.0000.5440). A amostra incluiu indivíduos de ambos os sexos com pé plano valgo flexível grave idiopático que foram submetidos à técnica calcâneo-stop em nossa instituição. A flexibilidade do pé foi avaliada com base na reversibilidade da deformidade com e sem teste de apoio de peso na ponta dos pés ( Fig. 1 ) e no resultado do teste de Jack.

**Fig. 1:** Teste de ficar na ponta dos pés para demonstrar a flexibilidade do pé. ( **A** ) À sustentação de peso, o arco medial colapsa. ( **B** ) O calcanhar se transforma em varo e o arco medial reaparece.

Os dados foram coletados de prontuários médicos, e a avaliação incluiu a resolução dos sintomas, a satisfação do paciente e de seus familiares, e parâmetros radiográficos pré e pós-operatórios. O estudo incluiu casos com documentação completa, assinatura do termo de consentimento livre e esclarecido, e cirurgias realizadas entre 2017 e 2022, com acompanhamento médio de 28 meses (variação: 12 a 65). Casos de desvio do eixo mecânico do membro inferior, deformidades ou rigidez concomitantes do pé, cirurgia ou fraturas prévias do pé, ou doenças locais ou sistêmicas subjacentes (como síndrome de Down, síndrome de Ehlers-Danlos etc.) foram excluídos.

## Procedimento Cirúrgico

Sob anestesia, com o joelho estendido, o pé foi forçado em dorsiflexão para avaliar o grau de valgo do calcâneo e verificar se havia associação a pé equino. Por palpação, identificou-se o seio do tarso, e uma incisão cutânea de aproximadamente 2,0 cm de comprimento foi feita sobre ele ( Fig. 2A ). A dissecção romba cuidadosa foi realizada para expor o coxim de gordura e o músculo extensor curto, assegurando que o ramo sensorial do nervo fibular permanecesse intacto. Sob fluoroscopia, o pé foi forçado em supinação, o que permitiu a identificação e a exposição da superfície plana na região lateral do calcâneo, que serviu como ponto de entrada para o seio do tarso. Um fio de Kirschner (K) de 2,0 mm de diâmetro foi inserido perpendicularmente na superfície óssea do calcâneo, fora do seio do tarso, e seu posicionamento foi confirmado à fluoroscopia ( Fig. 2B ). Posteriormente, o fio K foi removido, mas o retropé foi mantido em posição de varo. O ponto de entrada do fio foi identificado, o orifício foi ampliado usando uma broca de 3,2 mm, e um parafuso de aço inoxidável esponjoso de 6,5 mm com rosca parcial (DePuy Synthes, Raynham, MA, Estados Unidos), com comprimento entre 30 e 40 mm, foi inserido ( Fig. 2C,D ). A profundidade do parafuso foi ajustada para garantir que sua cabeça ficasse posicionada abaixo do processo lateral do tálus, corrigindo, assim, o desalinhamento em valgo do retropé. Em caso de sub ou supercorreção, o parafuso era respectivamente avançado ou retraído até alcançar o alinhamento desejado. Em alguns casos, implantes alternativos, como de parafusos corticais canulados de 7,0 mm (DePuy Synthes) ou parafusos de rosca completa, foram usados. Deve-se notar que a literatura disponível não indica nenhum impacto significativo dessas variações de implante nos resultados do procedimento. Se houvesse equino residual, um alongamento do tendão calcâneo do tipo Hoke era realizado, seguido da aplicação de uma tala de gesso de perna longa.

**Fig. 2:** Etapas cirúrgicas para a inserção do parafuso calcâneo. ( **A** ) Demarcação cutânea da incisão cirúrgica sobre o seio do tarso. ( **B** ) Após a dissecção romba, a entrada do seio do tarso foi exposta e um fio-guia de Kirschner foi inserido verticalmente no calcâneo. ( **C** ) Posicionamento do parafuso em vista lateral. ( **D** ) Posicionamento do parafuso em vista anteroposterior.

## Cuidado Pós-operatório

No pós-operatório, todos os pacientes foram imobilizados por 7 dias e, em seguida, a sustentação de peso foi gradualmente reintroduzida. Em casos que exigiram correção de equino, os pacientes usaram gesso para a caminhada por mais 2 semanas antes de fazer a transição para calçados regulares.

Não foi implementado um protocolo padronizado de fisioterapia, nem recomendado o uso de órteses ou de calçados modificados. Cerca de 3 semanas após a cirurgia, com a cicatrização completa, os pacientes foram orientados a retomar gradualmente as atividades físicas e os esportes, desde que a dor pós-operatória tivesse diminuído.

## Avaliação Clínica

O prontuário médico do paciente foi acessado para comparar os achados pré e pós-operatórios. Complicações decorrentes do procedimento cirúrgico, juntamente com sintomas observados no pós-operatório que não estavam presentes no pré-operatório, foram considerados, como dor, desconforto e melhora estética. 22

## Avaliação Radiográfica

As imagens pré e pós-operatórias mais recentes em incidências anteroposterior e de perfil, sob carga, foram avaliadas quanto aos seguintes ângulos e medidas: Meary, *pitch* calcâneo, Moreau-Costa-Bertani, alinhamento entre o eixo longo do tálus e o primeiro metatarso, cobertura angular talonavicular e cobertura percentual talonavicular. 23 24 A Fig. 3 mostra os ângulos usados, como traçá-los e os intervalos de referência. Todos os dados coletados foram agrupados e apresentados na Tabela 1 .

**Tabela 1:** Dados da amostra do estudo

Identificação	Idade	Acompanhamento	*Pitch* calcâneo		Ângulo de cobertura talonavicular		Porcentagem de cobertura talonavicular		Ângulo de Meary		Ângulo entre o tálus e o primeiro metatarso		Ângulo de Moreau-Costa-Bertani		Dor		Complicações
	(anos)	(meses)	Pré	Pós	Pré	Pós	Pré	Pós	Pré	Pós	Pré	Pós	Pré	Pós	Pré	Pós	
F1	9	20	4,5	11,8	45,6	22,4	50,6	24,8	-32,6	-5,5	25,3	2,7	159	140,8	Sim	Não	Não
F2	9	20	7,7	14	44,8	21,8	47,7	24,2	-23	-5,6	14,3	7,4	148,2	135,3	Sim	Não	Não
F3	13	34	6	16,7	36,6	16,5	40,6	18,3	-24	-13,7	12,4	3,4	142,4	132,4	Sim	Não	Não
F4	13	28	8,6	17,3	23,9	11,6	26,5	12,8	-22	-8,6	13,3	1,4	142,8	127,8	Sim	Não	Não
F5	9	34	2	4	56,1	24,7	62,3	27,4	-18,1	1,7	18,6	9,7	143,4	135	Sim	Não	Não
F6	9	34	-4	1,5	45,9	25	51	27,7	-18	-4,6	20,3	1,2	147,6	135,8	Sim	Não	Não
F7	11	40	2,6	10,5	45,8	18,7	50,8	20,7	-17,2	-0,8	17,1	5,5	146,1	133,1	Sim	Não	Dor crônica
F8	11	40	1	8,7	44,1	15,1	49	16,7	-22	-1,5	16,4	5	141,6	134,9	Sim	Sim	Dor crônica
F9	12	30	0,5	4,3	41,3	1	45,8	1,1	-17,8	-12,8	16,8	5,2	144,2	134	Sim	Não	Não
F10	12	30	0,5	6	50,4	23,3	56	25,8	-23,2	-9,8	36,1	17,3	151,5	135,3	Sim	Não	Não
F11	12	37	10,5	13,8	16	11,3	17,7	12,5	-6,7	6,3	25	14,3	130,3	115,6	Sim	Não	Não
F12	12	37	10,8	12,1	39,5	16,8	43,8	18,6	-14,6	1,6	36,2	19	134,7	119,5	Sim	Não	Não
F13	11	21	6,3	9,5	39	21,5	43,3	23,8	-30,6	-12,5	23,5	3	151,7	142,5	Sim	Não	Não
F14	11	21	9	7,8	11,9	9,8	13,2	10,8	-12,5	-7,7	6	7,8	141,6	137,7	Sim	Não	Não
F15	11	39	11	18	40,2	1,1	44,6	1,2	-10,7	13,3	24,1	6,5	138,7	118,6	Sim	Não	Não
F16	11	39	11,6	18,4	42,9	2,3	47,6	2,5	-12,5	-3,5	29,4	3,5	140	127	Sim	Não	Não
F17	12	17	4,7	5,1	32,9	20,2	36,5	22,4	-22,1	-13,5	12	12,7	152,6	144,2	Sim	Não	Subcorreção
F18	12	17	3,3	9,6	43	27,6	47,7	30,6	-28	-4,5	25,5	1,9	153,4	131,2	Sim	Não	Não
F19	11	47	9,6	15	38,8	12,8	43,1	14,3	-19	-5,3	19,2	9,3	151,2	129,5	Sim	Não	Não
F20	11	47	6,5	16,8	32	11,9	35,5	13,2	-19,7	-2	22,4	12,9	148,1	129,9	Sim	Não	Não
F21	9	65	3,9	17,3	42,8	15,5	47,5	17,2	-20,6	-4,4	16,8	8,6	144,8	130,4	Sim	Não	Não
F22	9	65	3,3	13,3	46,9	22,7	52,1	25,2	-30	-7,1	20,1	8,1	158	137,2	Sim	Não	Não
F23	9	6	3,6	7,9	26	20,4	28,8	22,6	-22,4	-12,2	4,4	2,3	148,4	141	Sim	Sim	Dor crônica
F24	16	33	10,1	12,2	35,6	31,7	39,5	35,2	-9,5	-6	14,8	13	131,4	132	Sim	Não	Não
F25	16	33	8,1	14,1	30,1	24,8	33,4	27,5	-14,6	-5,2	16	11	136,9	128,1	Sim	Não	Não
F26	11	32	7,9	15,6	41,6	15,1	46,2	16,7	-18,7	-5,7	13,6	1,1	142,9	137,6	Sim	Não	Não
F27	11	32	10,6	14,8	39,6	6,9	44	7,6	-13,4	-6,5	13,5	1,6	140,3	137,4	Sim	Não	Não
F28	7	11	7	12,7	41,6	7,1	46,2	7,8	-9,5	10,6	12,5	9,2	140,9	124,6	Sim	Não	Não
F29	7	11	6,7	8,2	33,8	4,5	37,5	5	-10,5	10,1	14,1	14,4	138,7	128	Sim	Não	Não
F30	13	26	13,4	20,1	32,5	12,1	36,1	13,4	-12,9	4,8	36,6	16	134,8	121,8	Sim	Não	Não
F31	13	26	19,3	20,6	36,4	16,6	40,4	18,4	-11,4	4,1	35,3	12,7	124,7	117,2	Sim	Não	Não
F32	10	6	9,7	16,2	26,4	14,7	29,3	16,3	-9,6	0,8	13,7	11,2	133,5	122,1	Sim	Não	Não
F33	10	6	14,4	17,3	20,8	2,3	23,1	2,5	-10,1	1,5	10,2	5	127,6	118	Sim	Não	Não
F34	11	47	5,7	8,1	27,7	23,4	30,7	26	-17,7	3,5	16	11,8	142,2	128,7	Sim	Não	Não
F35	11	47	7,1	8,7	23,8	18	26,4	20	-18,7	3,9	14	7	143,8	127,1	Sim	Não	Não
F36	13	20	10,4	17,4	25,2	9,9	28	11	-11,2	-1	4,2	4,7	133,2	119,1	Sim	Não	Não
F37	13	20	11,8	18,2	25,8	15,5	28,6	17,2	-13,6	-2,5	3,2	0,1	136,8	124,3	Sim	Não	Não
F38	12	12	3	12,7	43	23,9	47,7	26,5	-24	-3,5	22	9,8	158	126,5	Sim	Não	Não
F40	14	12	4,3	7,5	32,1	4,4	35,6	4,8	-21,7	-14,3	19,1	6,8	154,2	147,5	Sim	Não	Subcorreção
F41	14	12	5,5	11,1	36,7	5	40,7	5,5	-19,2	-10,4	23,2	9,8	150,1	143,2	Sim	Não	Subcorreção
F42	7	26	3,3	9,2	42	16,2	46,6	18	-13,3	-7,1	14,4	4,4	144,3	134,4	Sim	Não	Não
F43	7	26	4,1	10,1	40,7	13,3	45,2	14,7	-11,9	-6,6	13,2	3,9	142,1	129,1	Sim	Não	Não
F44	12	34	2,2	16,4	41,2	20,2	45,7	22,4	-23,1	-9,4	16,8	4,4	151,1	139,1	Sim	Não	Não

**Abreviaturas:** Pré, pré-operatório; Pós, pós-operatório.

**Fig. 3:** Avaliação radiográfica das deformidades do pé. ( **A** ) Ângulo de Meary: medido em vista lateral, representa o ângulo formado entre o eixo longo do tálus e o eixo longo do primeiro metatarso (intervalo de referência: 5°–15°). ( **B** ) *Pitch* calcâneo: medido em vista lateral, representa o ângulo formado entre a superfície inferior do córtex plantar do calcâneo e o solo (intervalo de referência: 15°–25°). ( **C** ) Ângulo de Moreau-Costa-Bertani: determinado traçando-se uma linha da superfície inferior do sesamoide medial até a superfície inferior da articulação talonavicular. Outra linha é traçada deste ponto até a tuberosidade posterior do calcâneo (intervalo de referência: 115°–125°). ( **D** ) Ângulo tálus-metatarso: medido em vista anteroposterior, representa o ângulo entre o eixo longo do tálus e o eixo longo do primeiro metatarso (intervalo de referência: 0°–5°). ( **E** ) Ângulo de cobertura da articulação talonavicular: medido em vista anteroposterior, representa o ângulo formado pelas superfícies articulares medial e lateral do tálus e a superfície articular do osso navicular (intervalo de referência: 0°–7°). ( **F** ) A cobertura percentual da cabeça do tálus é a porcentagem da superfície articular da cabeça do tálus coberta pelo navicular (normal: ≥ 90%).

## Análise Estatística

O teste de Shapiro-Wilk avaliou a normalidade da distribuição da amostra e determinou uma distribuição paramétrica, com significância estatística definida como *p* < 0,05. O teste *t* pareado foi utilizado para avaliar a significância da intervenção cirúrgica e a melhora dos resultados radiológicos, com *p* definido como < 0,001. As análises foram conduzidas usando o software RStudio Desktop Pro (Posit, PBC, Boston, MA, Estados Unidos), versão 2023.03.0.

## Resultados

Um total de 23 pacientes (44 pés), com idades entre 7 e 13 (média: 11) anos, foram considerados elegíveis para o estudo. O tempo mínimo de acompanhamento foi de 12 meses, e o máximo, de 5 anos (65 meses), com média de 28 meses.

Os resultados clínicos foram satisfatórios, tanto em termos das questões estéticas quanto do alívio da dor. Ao todo, 90% dos pacientes relataram melhora ou resolução completa dos sintomas e alívio da dor em comparação ao período pré-operatório. A taxa de complicações foi de 13,6%, e elas ocorreram em 6 casos: 3 com dor crônica no sítio cirúrgico e 3 com correção incompleta da deformidade. Em dois casos, a dor foi resolvida com injeções locais de corticosteroides e, no outro caso, o parafuso foi removido, o que diminuiu a dor, mas não a eliminou por completo. A remoção do implante não foi necessária nos outros pacientes. Não houve casos de infecção, problemas de cicatrização de feridas ou fratura do implante. Todos os parâmetros radiográficos exibiram melhora pós-operatória significativa ( Fig. 4 ).

**Fig. 4:** Aspecto clínico pré ( **A,B** ) e pós-operatório (C,D) de um menino de 10 anos submetido a cirurgia para a correção de pé plano valgo grave. Houve melhora significativa tanto no alinhamento do tornozelo quanto no arco do pé. Neste caso, além do procedimento de calcâneo-stop, foi realizado um alongamento percutâneo do tendão calcâneo para a correção de equino. ( **E** ) Radiografia pré-operatória em perfil tirada em 2019; ( **F** ) Radiografia pós-operatória em perfil tirada em 2022.

O *pitch* calcâneo apresentou aumento significativo, de 87%, com média pré-operatória de 6,61° (desvio-padrão [DP]: ± 0,65°) e pós-operatória de 12,41° (DP: ± 0,60°) ( Fig. 5 ). A cobertura talonavicular apresentou melhora significativa, de 136%, conforme evidenciado pelo teste *t* ( *p* < 0,001). Os valores médios finais de 15,43° (DP: ± 1,15°) permaneceram ligeiramente superiores aos valores de referência ( Fig. 6 ). O ângulo de Meary apresentou forte correlação no teste t ( *p* < 000,1) ( Fig. 7 ) com melhora significativa de 383%, no alinhamento talonavicular do eixo sagital; entretanto, o valor final médio, de -3,64° (DP: ± 1,01°), ainda não estava dentro da faixa normal.

**Fig. 5:** Distribuição em diagrama de caixa do ângulo de *pitch* calcâneo pré e pós-operatório. Houve um aumento pós-operatório significativo, e o ângulo atingiu valores normais ( *p* < 0,001).

**Fig. 6:** Distribuição em diagrama de caixa do ângulo talonavicular pré e pós-operatório. Houve uma melhora significativa no pós-operatório, e o ângulo quase atingiu valores normais ( *p* < 0,001).

**Fig. 7:** Distribuição em diagrama de caixa do ângulo de Meary pré e pós-operatório. Houve uma melhora significativa no pós-operatório, e o ângulo ficou ligeiramente abaixo dos valores normais ( *p* < 0,001).

O alinhamento do eixo longo do tálus com o primeiro metatarso no plano coronal apresentou melhora de 141%, com diferença significativa no teste *t* ( *p* < 0,001) ( Fig. 8 ). O valor médio pré-operatório foi de 18,12° (DP: ± 1,22°), e o pós-operatório, de 7,5° (DP: ± 0,78°).

**Fig. 8:** Distribuição em diagrama de caixa do ângulo tálus-metatarso pré e pós-operatório. Houve uma melhora significativa após a cirurgia, e o ângulo quase atingiu valores normais ( *p* < 0,001).

O ângulo de Moreau-Costa-Bertani apresentou melhora ( *p* < 0,001). Apesar de os valores não terem atingido a normalidade, com média de 143,8° (DP: ± 1,22°) no pré-operatório e de 131,21° (DP: ± 1,19°) no pós-operatório, observou-se melhora de 9% na formação do arco plantar ( Fig. 9 ). A porcentagem de cobertura talonavicular apresentou melhora significativa, de 38%, conforme evidenciado pela análise do teste t ( *p* < 0,001). O valor médio pré-operatório, de 59,7% (DP: ± 1,54%) aumentou para 82,8% (DP: ± 1,28%), com melhora significativa na relação entre o retropé e o mediopé ( Fig. 10 ).

**Fig. 9:** Distribuição em diagrama de caixa do ângulo de Moreau-Costa-Bertani pré e pós-operatório. Houve um aumento pós-operatório significativo, e o ângulo atingiu valores normais ( *p* < 0,001).

**Fig. 10:** Distribuição em diagrama de caixa da porcentagem de cobertura talonavicular pré e pós-operatória. Houve uma melhora significativa no pós-operatório, e a porcentagem quase atingiu valores normais ( *p* < 0,001).

## Discussão

Em nossa série de casos, o tratamento do pé plano pediátrico idiopático grave usando a técnica calcâneo-stop demonstrou correção eficaz da deformidade com complicações mínimas. O procedimento cirúrgico é tecnicamente simples e produz resultados clínicos e radiográficos positivos.

Embora a causa exata do pé plano continue a ser debatida, 7 25 a ausência de tratamento pode levar a limitações funcionais na idade adulta. Pode haver o desenvolvimento de subluxação progressiva e carga excessiva do complexo articular triplo e do ligamento de mola, o que, em última análise, leva à degeneração articular tardia. Essa sobrecarga é clinicamente responsável por fadiga, dor e insuficiência mecânica.

A técnica extra-articular conhecida como calcâneo-stop envolve a inserção de um parafuso no calcâneo, que bloqueia de modo eficaz a pronação excessiva do tálus-calcâneo e as subluxações subsequentes. 17 Além disso, pesquisas 15 26 27 sugerem que, além da restrição articular, os mecanismos proprioceptivos influenciam a cinética da marcha. Diferentemente das osteotomias, que alteram imediatamente as relações articulares e a distribuição de carga, a artrorrise induz uma correção precoce das deformidades, seguida de uma reorganização articular tridimensional gradual e tardia ao longo de vários meses, o que leva à remodelação articular.

As osteotomias de alongamento da coluna lateral e de medialização do calcâneo são tratamentos aceitos para o pé plano flexível sintomático em indivíduos imaturos, com resultados satisfatórios relatados em aproximadamente 90% dos casos. 8 9 10 No entanto, esses procedimentos são mais invasivos, exigem maior tempo de recuperação, e impõem restrições de atividade por semanas. Por outro lado, a reabilitação da artrorrise é rápida, o que permite que o paciente ande logo após a cirurgia. 13 17 27 28 Ainda assim, em nossa série, 42% dos pacientes foram submetidos ao alongamento do tendão calcâneo e necessitaram de imobilização gessada por até 21 dias. Nos demais pacientes, a descarga de peso foi permitida a partir do sétimo dia de pós-operatório. Alguns autores, 15 porém, não liberam rotineiramente o tríceps sural, mesmo na presença de equino, argumentando que o equino pós-operatório pode ser revertido com fisioterapia.

Embora exista uma dissociação clínico-radiográfica, empregamos parâmetros angulares para avaliar e melhorar a objetividade e a reprodutibilidade dos resultados. No entanto, essas variáveis podem não estar diretamente correlacionadas aos sintomas e à gravidade do pé. Além disso, estabelecer os sinais mais significativos para o diagnóstico e determinar o limite preciso para definir um pé como plano continuam a ser desafios. A relevância das medidas radiográficas e do diagnóstico de pé plano ainda está em debate. 23

Nossas avaliações radiográficas revelaram melhora nos parâmetros do retropé e do médiopé ao longo de um período médio de acompanhamento de 28 meses, consistente com os achados de estudos anteriores. 19 27 28 Os desfechos clínicos foram considerados satisfatórios, pois 90% dos pacientes relataram ausência de dor. A taxa de complicações foi de 13,6%, mas elas foram de baixa gravidade, atribuídas principalmente à dor, com melhora após tratamento analgésico. Nossos dados e taxas de complicações são semelhantes aos relatados em outros estudos. 19 20 27

A determinação do momento ideal para a remoção do implante sem comprometimento da correção ainda é incerta na técnica calcâneo-stop. Alguns autores 11 29 sugerem a remoção após 2 anos, período em que ocorre a remodelação articular e a incorporação do reflexo proprioceptivo. Porém, estabelecer um protocolo de remoção em nosso estudo foi difícil, devido às altas demandas de serviços hospitalares. Em decorrência disso, a maioria dos pacientes manteve o implante por até 5 anos sem apresentar hipercorreção, dor ou fratura. As imagens radiográficas não mostraram evidências de qualquer impressão no tálus secundária ao contato prolongado na interface do implante, mas não avaliamos precisamente esse parâmetro devido aos sintomas limitados.

Nosso estudo tem limitações, incluindo o tamanho restrito da amostra. Além disso, enfrentamos desafios para quantificar a natureza subjetiva e variável da dor e do desconforto em uma população pediátrica e estabelecer uma correlação entre os parâmetros radiográficos e os achados clínicos do pé plano.

Nossa impressão geral é a de que a técnica calcâneo-stop parece ser uma opção adequada para o tratamento do pé plano flexível sintomático em jovens de acordo com os principais estudos. 30 31 Esta técnica pode corrigir a deformidade inicial e melhorar de forma consistente os parâmetros clínicos e radiográficos. Além disso, a técnica pode ser revertida por meio da retirada do parafuso, se necessário, e não compromete o uso de outras técnicas em casos de falha. Ainda há dúvidas quanto à remodelação articular e às possíveis repercussões no longo prazo; no entanto, a técnica parece promissora.
